# A FAERS database study on visual impairment adverse events associated with three long-acting insulin analogues

**DOI:** 10.3389/fendo.2026.1750408

**Published:** 2026-03-17

**Authors:** Li Zhong, Wenxuan Cao, Xinyu Liu, Tiancai Wen, Jing Yan

**Affiliations:** 1 China Academy of Chinese Medical Sciences Data Center, Beijing, China; 2 China Academy of Chinese Medical Sciences Eye Hospital, Beijing, China

**Keywords:** adverse events, FAERS database, long-acting insulin analogues, pharmacovigilance, visual impairment

## Abstract

**Purpose:**

To utilize the U.S. Food and Drug Administration Adverse Event Reporting System (FAERS) to systematically evaluate and compare the risk signals, clinical characteristics, and potential influencing factors of visual impairment adverse events (AEs) associated with three major long-acting insulin analogues (insulin degludec, insulin detemir, and insulin glargine).

**Methods:**

This retrospective pharmacovigilance study analyzed FAERS data from Q1 2004 to Q2 2025. Reports explicitly listing any of the three target insulins as the “primary suspect” were extracted. Data extraction and deduplication were conducted in accordance with FDA guidelines. Disproportionality analysis was performed using the Reporting Odds Ratio (ROR), Proportional Reporting Ratio (PRR), Bayesian Confidence Propagation Neural Network (BCPNN), and Multi-item Gamma Poisson Shrinker (MGPS). Furthermore, multivariate regression and Weibull distribution analyses were employed to identify potential clinical risk factors and time-to-onset patterns of these ocular events.

**Results:**

A total of 102,953 primary suspect reports were analyzed. Statistically significant visual impairment signals were detected across all three insulins. Insulin glargine exhibited the strongest overall association (ROR = 3.60, 95% *CI*: 3.54–3.67), whereas insulin degludec demonstrated the weakest (ROR = 1.59, 95%*CI*: 1.47–1.72). Regarding specific event types, insulin degludec and insulin detemir were primarily associated with diabetic retinopathy, whereas insulin glargine showed the strongest association with diabetic glaucoma (ROR = 50.36). Among the 5,780 specific “visual impairment” reports, over 60% involved female patients, and 93% originated from the United States. Multivariate regression suggested that higher body weight might be a potential protective factor (P*<*0.05); however, this finding may be confounded by the database’s inherent limitation in differentiating between Type 1 and Type 2 diabetes characteristics. Weibull analysis confirmed an early-onset pattern (early failure model) for all three insulins, with insulin degludec showing the shortest median time-to-onset (23.6 days).

**Conclusions:**

Long-acting insulins are associated with varying risks of visual impairment AEs, exhibiting an early-onset temporal pattern. Insulin glargine demonstrated stronger disproportionality signals for glaucoma, while degludec and detemir were more closely associated with retinopathy. These characteristics suggest the necessity of implementing individualized ophthalmic monitoring during the early phase of initiating basal insulin therapy in diabetic patients.

## 1 Introduction

Diabetes mellitus is a globally prevalent chronic metabolic disease. Its incidence continues to rise, making it a significant public health challenge. According to recent epidemiological statistics from the International Diabetes Federation (IDF), the global diabetic population has exceeded 537 million (1). and is projected to reach 783 million by 2045. Diabetes and its associated systemic complications significantly reduce patients’ quality of life and impose substantial economic and clinical burdens on healthcare systems worldwide. Among these complications, ocular morbidities—particularly diabetic retinopathy, macular edema, and secondary glaucoma—are leading causes of preventable blindness in working-age adults.

Long-acting insulin analogs (insulin degludec, insulin detemir, and insulin glargine) are core basal medications for glycemic control in clinical practice. They are designed to mimic physiological basal insulin secretion, thereby maintaining stable fasting blood glucose levels. However, post-marketing surveillance data from the FAERS database and clinical observations suggest potential safety signals, indicating that the initiation of these medications may be associated with visual impairment adverse events (AEs). This clinical phenomenon prompts further investigation: while insulin is utilized to prevent hyperglycemia-induced microvascular damage, its use during certain specific phases may be correlated with the onset of visual disturbances.

The pathological risk of visual impairment in this patient population is multifactorial. Primarily, it is driven by the chronic hyperglycemic state inherent to diabetes. Prolonged local hyperglycemia damages the ocular microenvironment through multiple cascading pathways, including the activation of the polyol pathway, the acceleration of advanced glycation end products (AGEs) formation, and the release of reactive oxygen species (ROS). These mechanisms collectively compromise the blood-retinal barrier, laying the pathological foundation for visual impairment.

Beyond the progression of the disease itself, the differences in risk are considered to be associated with the distinct pharmacological profiles of the three long-acting insulin analogs. These medications exhibit significant differences in molecular structure, isoelectric points, subcutaneous absorption stability, and binding affinities for insulin-like growth factor-1 (IGF-1) receptors. These varying characteristics may uniquely interfere with the homeostasis of ocular tissues, leading to the differentiation in the risk and type of visual impairment AEs (2–5).

Despite these clinical concerns, existing literature exhibits certain limitations regarding the comparative ocular safety of these drugs. Most current studies have primarily focused on the short-term visual symptoms associated with a single insulin analogue, lacking a comprehensive head-to-head comparative analysis among the three mainstream basal insulins. For instance, in a 5-year randomized open-label study, Rosenstock et al. (6)compared retinopathy risks between insulin glargine and neutral protamine Hagedorn (NPH) insulin but did not include insulin detemir and degludec in their scope. Similarly, although Larose et al. (7) conducted a large-cohort study using the UK Clinical Practice Research Datalink (CPRD) to evaluate long-acting insulins as a whole, they did not sub-classify the individual risks associated with specific medications. Furthermore, the clinical risk factors, demographic influences, and exact time-to-onset patterns of visual impairment remain to be fully elucidated, resulting in a lack of reliable, evidence-based reference for ophthalmic monitoring during the initiation of insulin therapy.

To fill this knowledge gap, this study adopted a retrospective pharmacovigilance design utilizing real-world data from the FAERS database spanning from Q1 2004 to Q2 2025. By applying disproportionality analysis, multivariate regression, and Weibull distribution, this study aims to systematically evaluate and compare the occurrence characteristics, signal strengths, specific event phenotypes, and temporal patterns of visual impairment AEs associated with insulin degludec, detemir, and glargine. The findings aim to provide a scientific basis for the rational prescription of basal insulins and offer references for visual health management in patients with diabetes.

## 2 Methods

### 2.1 Data collection

FAERS is currently one of the largest publicly accessible post-marketing safety monitoring databases for pharmaceuticals and biological products. Managed by the FDA’s MedWatch program, it collects spontaneous adverse event reports submitted by healthcare professionals, consumers, and pharmaceutical manufacturers (8). Because FAERS is a publicly accessible database containing fully anonymized patient data, this study was exempt from institutional review board (IRB) ethical approval.

To conduct a multidimensional analysis, this study utilized relevant modules within the FAERS database from Q1 2004 to Q2 2025. The DEMO module provided basic patient demographic data (e.g., age, gender, weight) for baseline characterization. The DRUG and INDI modules were utilized to identify reports explicitly listing the target long-acting insulins as the “primary suspect” (PS) and to confirm their indicated use. Standardized adverse event terms related to ocular issues were extracted from the REAC module, which employs the Medical Dictionary for Regulatory Activities (MedDRA) Preferred Terms (PTs). The OUTC module assisted in assessing the severity and outcome of events, while the THER module supplied the specific dates of drug administration and AE onset, which were used to calculate the time-to-onset patterns.

### 2.2 Data extraction and deduplication

Data extraction and cleaning were executed in accordance with FDA-recommended guidelines to ensure dataset standardization. Considering that the FAERS database frequently contains updated and follow-up reports for the same case, raw data were sorted utilizing three core fields from the demographic table: primaryid, caseid, and FDA_DT (FDA receive date) to obtain the “latest case version.” Specifically, duplicate records with identical caseid and FDA_DT values were deleted. For instances with the same caseid, only the entry bearing the highest (i.e., most recent) primaryid value was retained. Concurrently, any reports officially labeled as “deleted” by the FDA were excluded.

**Figure 1:**
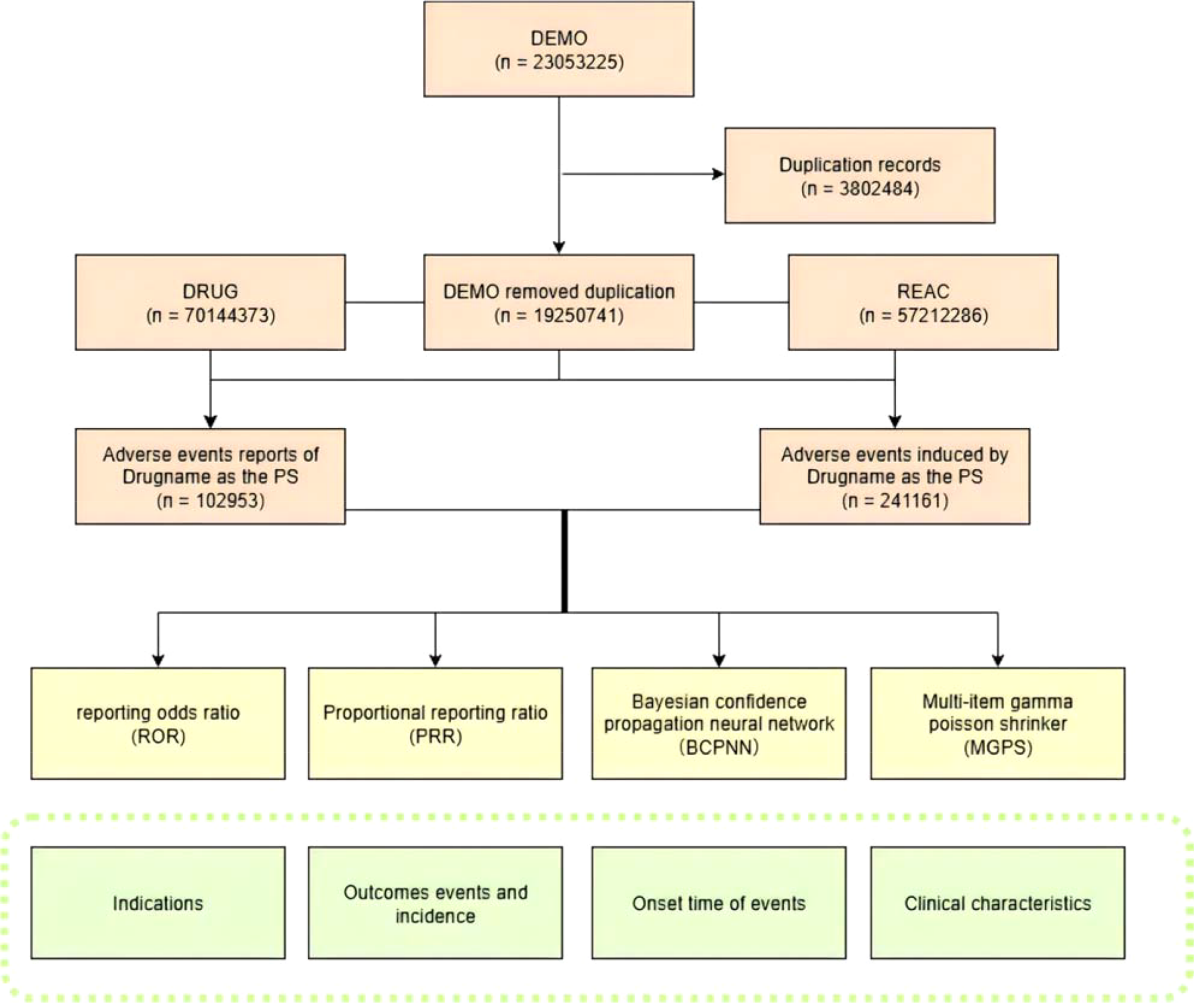
Flowchart of patient selection and data extraction from the FAERS database.

Following deduplication, records lacking critical clinical information (e.g., missing drug exposure dates or incomplete adverse event descriptions) were excluded. Only reports explicitly designating insulin degludec, insulin detemir, or insulin glargine as the primary suspect (PS) were included in the final analytical cohort. The detailed data selection process is illustrated in **Figure 1**.

### 2.3 Target drug screening and event standardization

This study used standardized active pharmaceutical ingredients and their common international trade names to identify the target drugs. For insulin detemir, search terms included “Insulin Detemir”, “Levemir”, “Levemir FlexPen”, and “Levemir PenFill”. For insulin degludec, terms such as “Tresiba” and “Insulin Degludec” were utilized. For insulin glargine, the search strategy incorporated “LANTUS SOLOSTAR”, “Insulin Glargine”, and “Lantus+Basaglar”.

Adverse events were standardized using MedDRA PTs. Initially, 19 distinct ocular-related PTs were screened to evaluate the comprehensive ophthalmic safety profile of the three drugs. In subsequent multivariate regression and time-to-onset analyses, the specific PT “VISUAL IMPAIRMENT” was selected as the primary clinical functional endpoint. This approach was adopted to reduce the pathological heterogeneity caused by pooling anatomical lesions (e.g., cataracts) with functional deficits, thereby allowing for a more focused assessment of risk factors directly affecting patient vision.

### 2.4 Statistical analysis

This study employed four commonly used disproportionality analytical methods to quantify the signal strength of drug-associated ocular adverse events (9). These included two frequentist methods—the Reporting Odds Ratio (ROR) (**Table 1**) (10)and the Proportional Reporting Ratio (PRR) (11),—and two Bayesian methods—the Bayesian Confidence Propagation Neural Network (BCPNN) (12), and the Multi-item Gamma Poisson Shrinker (MGPS). The combined use of multiple algorithms helps to reduce the inherent statistical biases of a single method and improves the reliability of the detected safety signals.

**Table 1:** Fourfold table for ROR calculation and overall ADE signal results.

Type of drug	Target adverse reaction reports	Other adverse reaction reports	Sum
Target drug	*a*	*b*	*a+b*
Other drug	*c*	*d*	*c+d*
Sum	*a+c*	*b+d*	*n=a+b+c+d*

### 2.5 Criteria for signal determination

A signal was considered statistically significant when it met the following established thresholds:

1. ROR Method: The lower limit of the 95% confidence interval (CI) for the ROR is > 1.
2. PRR Method: The PRR is ≥ 2, accompanied by a Chi-square (χ²) value ≥ 4.
3. BCPNN Method: The lower limit of the 95% CI for the Information Component (IC025) is > 0.
4. MGPS Method: The lower limit of the 95% CI for the Empirical Bayes Geometric Mean (EBGM05) is > 1.

## 3 Results

### 3.1 Signal detection of overall adverse drug events

**Figure 2:**
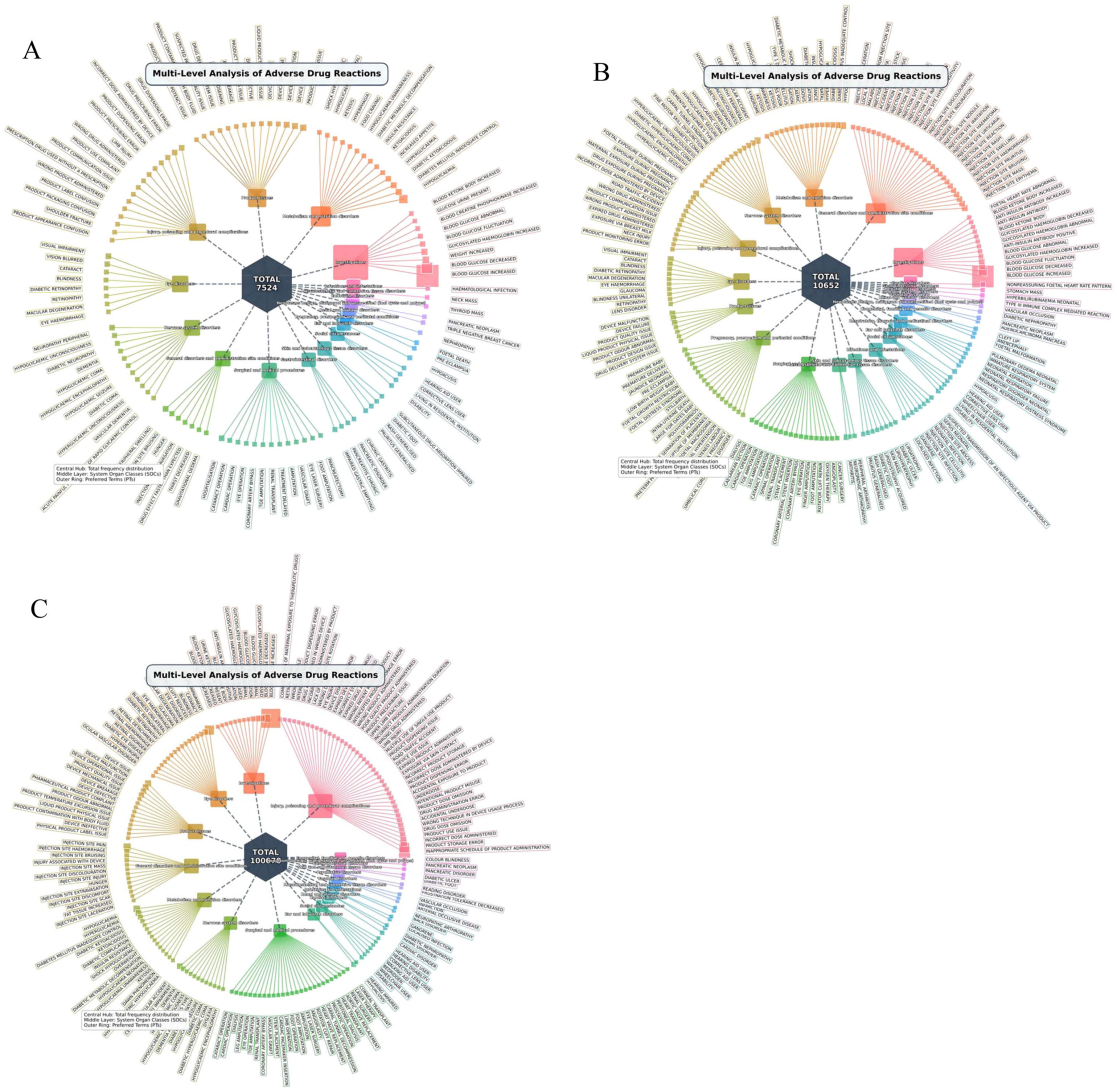
System organ class (SOC) involvement in adverse drug event (ADE) signals **(A)** Insulin Degludec: multi-level SOC analysis (18 SOCs involved). System organ class (SOC) involvement in adverse drug event (ADE) signals **(B)** Insulin detemir: multi-level SOC analysis (23 SOCs involved); System organ class (SOC) involvement in adverse drug event (ADE) signals **(C)** Insulin glargine: multi-level SOC analysis (21 SOCs involved);.

This section evaluated the overall ADE association signals for insulin degludec (n=156 ocular-related ADEs), insulin detemir (n=249 ocular-related ADEs), and insulin glargine (n=5380 ocular-related ADEs). The results of the disproportionality analysis are detailed in **Table 2**, and a comparison of the significant signals among the three insulins is presented in **Figure 2**.

**Table 2:** Signal detection results of overall adverse drug events for three types of insulin.

Parameter	Insulin degludec (n = 156)	Insulin detemir (n = 249)	Insulin glargine (n = 5380)
ROR(95%Cl)	1.51 (1.39 - 1.63)*	2.10 (1.97 - 2.23)*	3.60 (3.54 - 3.67)*
PRR(χ2)	1.49 (100.95)	2.05 (602.18)*	3.43 (22943.90)*
EBGM(EBGM05)	1.49 (1.38)	2.05 (1.93)	3.40 (3.34)*
IC(IC025)	0.58 (0.46)*	1.04 (0.95)*	1.76 (1.74)*

”*” indicates that the confidence interval of the corresponding index does not include the null value (ROR, PRR, and EBGM take “1” as the null value, while IC takes “0” as the null value), suggesting that the association between adverse drug events and insulin is statistically significant (P<0.05).

ROR, Reporting Odds Ratio; PRR, Proportional Reporting Ratio; IC025, lower limit of 95% CI for Information Component; EBGM05, lower limit of 95% CI for Empirical Bayes Geometric Mean; CI, Confidence Interval.

The association indicators for all three basal insulins met the criteria of “ROR>1, EBGM05>1, IC025>0”, indicating statistically significant safety signals for ocular ADEs (P<0.05). These association strengths exhibited a certain hierarchical distinction: insulin glargine demonstrated relatively the strongest overall association signal across all metrics; insulin degludec showed the weakest overall association, while insulin detemir positioned intermediately. Furthermore, **Table 2** indicates that due to the larger reporting sample size of insulin glargine (n=5380), it generated a significantly higher χ² value (22943.90) in the PRR analysis compared to insulin detemir (602.18) and insulin degludec (100.95), a mathematical result that objectively enhanced its statistical power.

### 3.2 Specific ocular-related adverse reactions

**Figure 3:**
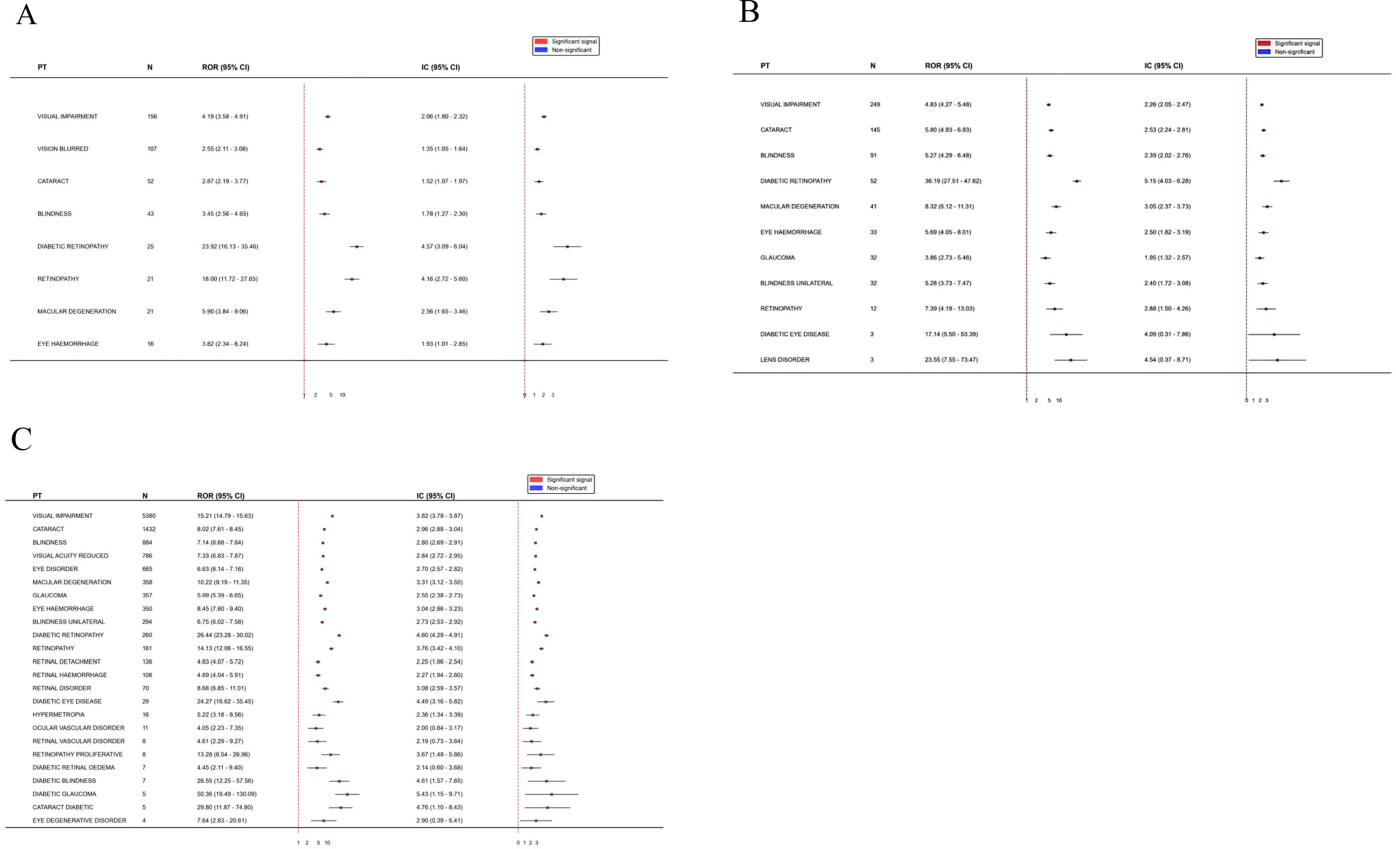
Three insulins: ROR and IC for eye disorders: **(A)** Insulin Degludec: reporting odds ratio (ROR) & BCPNN information component (IC) for eye disorders. Three Insulins: ROR and IC for Eye Disorders: **(B)** Insulin detemir: ROR & IC for eye disorders. Three Insulins: ROR and IC for Eye Disorders: **(C)** Insulin glargine: ROR & IC for eye disorders.

At the level of specific ocular system PTs, we further analyzed the differential phenotypes of visual AEs induced by the three insulins (**Table 3**). All 19 included ocular PTs (encompassing visual impairment, cataracts, diabetic retinopathy, etc.) met the criteria for significant signals (ROR>2, IC025>0, P<0.05) (**Figure 3**).

**Table 3:** Signal detection results of ocular adverse reactions related to three types of insulin.

Classification of PT	Insulin degludec (n = 156)	Insulin detemir (n = 249)	Insulin glargine (n = 5380)
Visual impairment			
ROR(95%Cl)	4.19 (3.58 - 4.91)	4.83 (4.27 - 5.48)	15.21 (14.79 - 15.63)
PRR(χ2)	4.17 (375.63)	4.80 (748.25)	14.81 (66084.38)
EBGM(EBGM05)	4.16 (3.56)	4.79 (4.23)	14.15 (13.76)
IC(IC025)	2.06 (1.80)	2.26 (2.05)	3.82 (3.78)
Cataract			
ROR(95%Cl)	2.87 (2.19 - 3.77)	5.80 (4.93 - 6.83)	8.02 (7.61 - 8.45)
PRR(χ2)	2.87 (63.33)	5.78 (571.76)	7.97 (8502.28)
EBGM(EBGM05)	2.87 (2.18)	5.76 (4.90)	7.78 (7.38)
IC(IC025)	1.52 (1.07)	2.53 (2.24)	2.96 (2.88)
Blindness			
ROR(95%Cl)	3.45 (2.56 - 4.65)	5.27 (4.29 - 6.48)	7.14 (6.68 - 7.64)
PRR(χ2)	3.44 (74.47)	5.26 (313.17)	7.11 (4537.72)
EBGM(EBGM05)	3.44 (2.55)	5.25 (4.27)	6.97 (6.52)
IC(IC025)	1.78 (1.27)	2.39 (2.02)	2.80 (2.69)
Macular degeneration			
ROR(95%Cl)	5.90 (3.84 - 9.06)	8.32 (6.12 - 11.31)	10.22 (9.19 - 11.35)
PRR(χ2)	5.89 (85.20)	8.31 (262.72)	10.20 (2870.49)
EBGM(EBGM05)	5.89 (3.83)	8.28 (6.09)	9.89 (8.90)
IC(IC025)	2.56 (1.65)	3.05 (2.37)	3.31 (3.12)
Eye haemorrhage			
ROR(95%Cl)	3.82 (2.34 - 6.24)	5.69 (4.05 - 8.01)	8.45 (7.60 - 9.40)
PRR(χ2)	3.82 (33.29)	5.69 (127.21)	8.44 (2231.31)
EBGM(EBGM05)	3.82 (2.34)	5.68 (4.03)	8.23 (7.40)
IC(IC025)	1.93 (1.01)	2.50 (1.82)	3.04 (2.86)
Diabetic retinopathy			
ROR(95%Cl)	23.92 (16.13 - 35.46)	36.19 (27.51 - 47.62)	26.44 (23.28 - 30.02)
PRR(χ2)	23.89 (543.87)	36.12 (1746.53)	26.40 (5828.01)
EBGM(EBGM05)	23.70 (15.99)	35.54 (27.01)	24.30 (21.40)
IC(IC025)	4.57 (3.09)	5.15 (4.03)	4.60 (4.29)
Retinopathy			
ROR(95%Cl)	18.00 (11.72 - 27.65)	7.39 (4.19 - 13.03)	14.13 (12.06 - 16.55)
PRR(χ2)	17.98 (334.82)	7.39 (66.05)	14.12 (1871.74)
EBGM(EBGM05)	17.88 (11.64)	7.37 (4.18)	13.51 (11.53)
IC(IC025)	4.16 (2.72)	2.88 (1.50)	3.76 (3.42)

ROR, Reporting Odds Ratio; PRR, Proportional Reporting Ratio; IC025, lower limit of 95% CI for Information Component; EBGM05, lower limit of 95% CI for Empirical Bayes Geometric Mean; CI, Confidence Interval.

**Figure 4:**
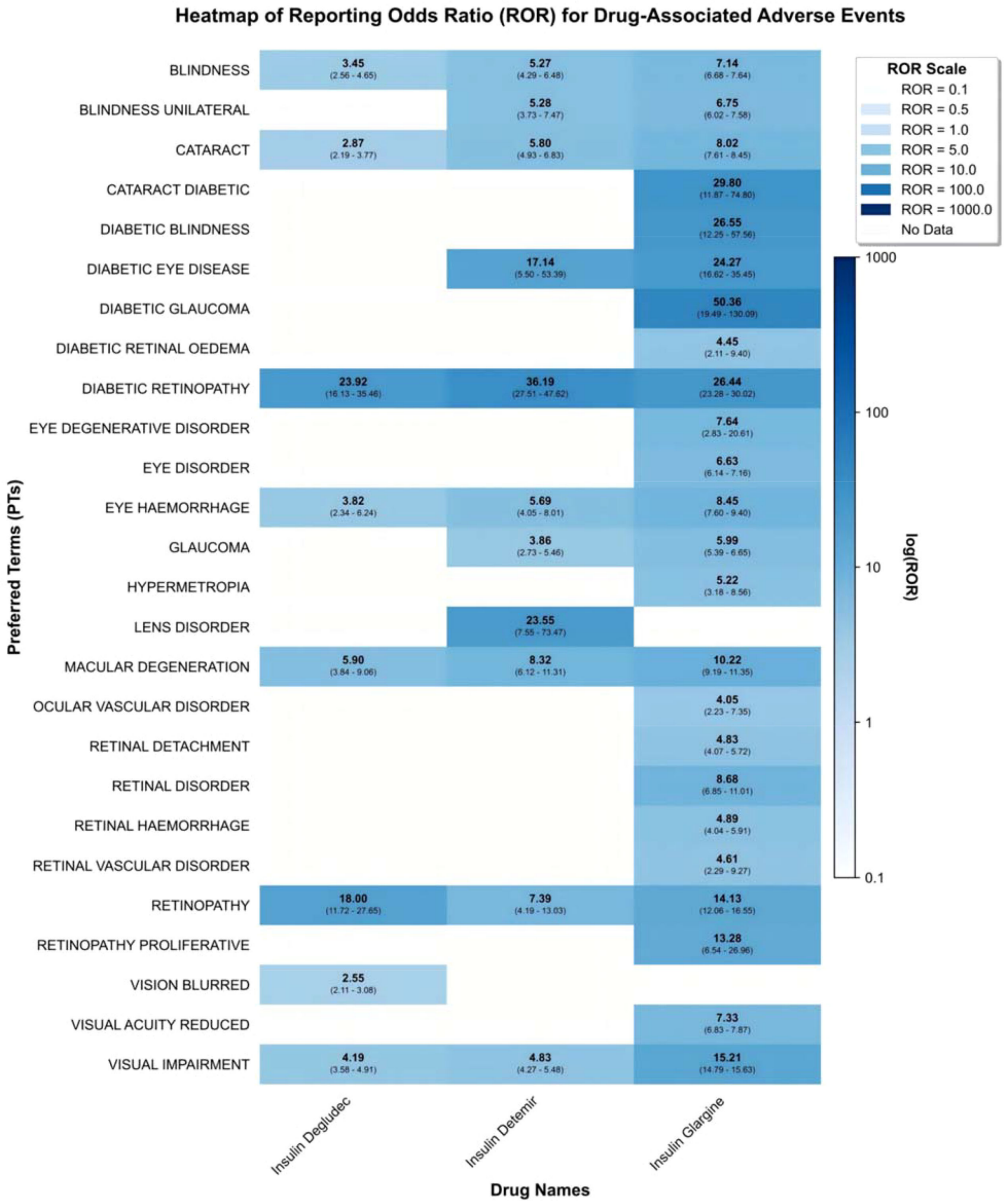
Distribution of the number of adverse events for eye disorders related to three types of long-acting insulin injections.

However, event specificity showed certain differences. Insulin degludec was primarily associated with 8 ocular PTs, among which diabetic retinopathy demonstrated the strongest association strength. Similarly, among the 10 ocular PTs linked to insulin detemir, diabetic retinopathy yielded the highest ROR. In contrast, insulin glargine, which possessed the largest sample size and involved all 19 ocular PTs, demonstrated the most prominent association with diabetic glaucoma (ROR = 50.36), with a signal strength higher than that for retinopathy. The distribution in **Figure 4** visually displays the number of specific ocular AE reports, illustrating the main proportion of insulin glargine across various ocular PT categories.

### 3.3 Basic characteristics and regression analysis

**Figure 5:**
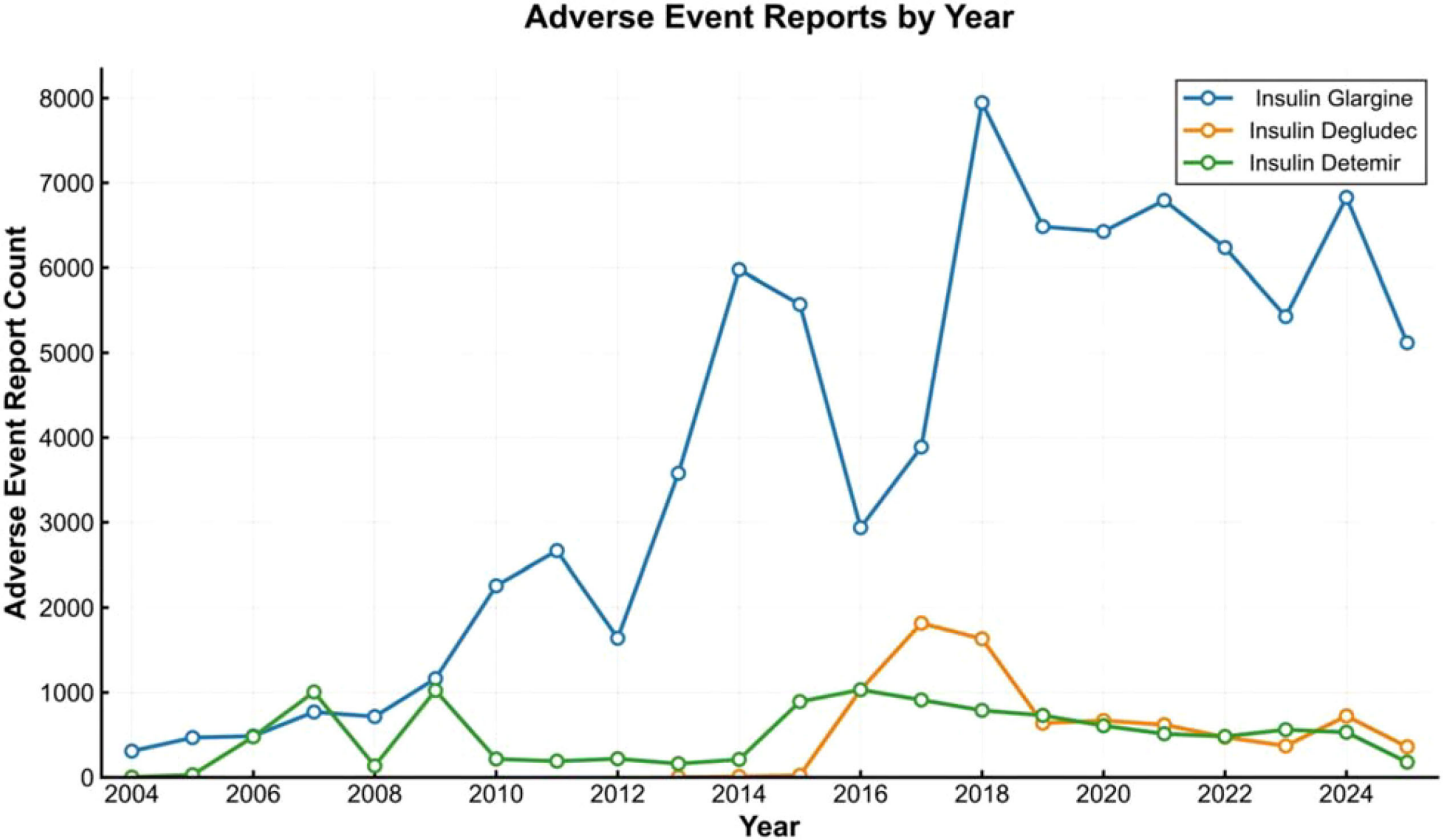
Frequency of adverse event reports related to eye disorders for three types of long - acting insulin injections from 2004 to 2025.

Given that visual impairment is a functional endpoint resulting from multiple ocular conditions, we selected “VISUAL IMPAIRMENT” as the primary PT to investigate related demographic risk factors. As shown in **Figure 5**, the number of the 5,780 visual impairment reports spanning Q1 2004 to Q2 2025 showed a fluctuating upward trend, with the upward trajectory of insulin glargine being particularly evident.

Demographic analysis (**Table 4**) revealed a higher proportion of female patients in the adverse event reports, accounting for 64.7%, 65.1%, and 64.7% of reports for degludec, detemir, and glargine, respectively. Geographically, the United States was the primary source, contributing over 93% of the reports across all three drugs.

**Table 4:** Summary of Basic Information on three types of long-acting insulin injections-Related ADE Reports.

Parameter	Insulin degludec (n = 156)	Insulin detemir (n = 249)	Insulin glargine (n = 5380)
Sex			
Female	101	162	3481
Male	53	82	1649
NA	3	5	250
Reporters’country			
United States	146	236	5173
Japan	0	0	2
United Kingdom	2	1	4
China	1	0	8
Others	7	0	193
Age group			
<18	0	0	1
18-64	28	54	1286
>65	41	70	2255
NA	87	125	1863
Reported year			
2008	0	0	3
2009	0	2	66
2010	0	0	72
2011	0	1	62
2012	0	3	71
2013	0	2	197
2014	0	0	325
2015	0	13	349
2016	5	26	330
2017	10	31	370
2018	7	22	440
2019	13	32	373
2020	24	22	547
2021	26	21	750
2022	16	20	496
2023	11	19	363
2024	34	31	351
2025	10	4	215

**Figure 6:**
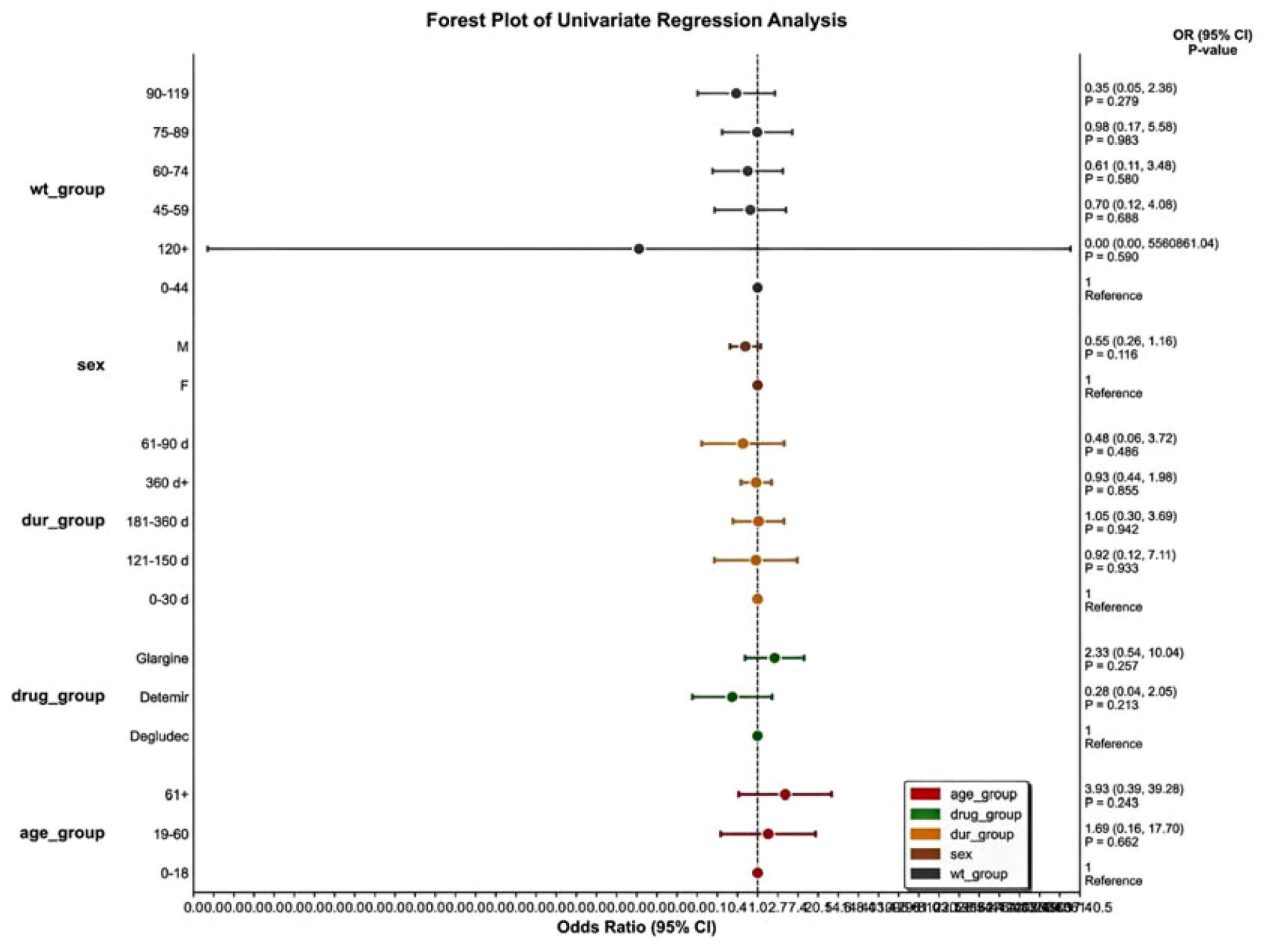
Multivariate regression analysis of three types of insulin injections based on VISUAL IMPAIRMENT (Compared with Insulin Degludec).

Using insulin degludec as the reference group, multivariate regression analysis was performed to identify potential influencing variables (**Table 4**, **Figure 6**). Results indicated that the weight group variable (wt_group) suggested a statistical association where higher body weight might correspond to a lower incidence of reported visual impairment, acting as a potential protective factor (P<0.05). However, it should be noted that body weight data in the FAERS database had an exceptionally high missing rate (missing in 88.4%, 76.8%, and 91.5% of reports for insulin degludec, detemir, and glargine, respectively); therefore, interpretation of this result requires extreme caution.

### 3.4 Time-to-onset pattern (Weibull distribution)

To evaluate the temporal dynamic characteristics of visual AEs, Weibull distribution analysis was conducted based on the time interval between therapy initiation and event onset. The results (**Table 5**) showed that the shape parameters (β) for all three insulins were less than 1, conforming to the “early failure pattern.” This indicates that the hazard rate of experiencing visual impairment is relatively high during the initial period following the initiation of insulin therapy and gradually decreases over time.

**Table 5:** Weibull Distribution situation of VISUAL IMPAIRMENT induced by three types of long-acting insulin injections.

Insulin type	Case (n)	TTO (Median)	TTO (Max-min)	Shape parameter(α)	95%CI (α)	Scale parameter(β)	95%CI (β)	Failure type
Insulin Degludec	156	23.6(1.0-219.8)	1-821	58.47	8.54-232.50	0.4	0.34-0.79	Early failure
Insulin Detemir	249	38.5(1.0-732.5)	1-4119	127.54	9.64-860.40	0.31	0.27-0.48	Early failure
Insulin Glargine	5380	175.1(2.8-1177.5)	1-5841	401.13	287.53-596.76	0.44	0.40-0.51	Early failure

The median time-to-onset for insulin degludec was relatively short at 23.6 days, followed by insulin detemir at 38.5 days. Insulin glargine exhibited a comparatively longer median onset time of 175.1 days. Notably, 50% of the reported visual impairment cases for degludec and detemir occurred within the first 1.3 and 5.2 months, respectively, suggesting that the early phase of treatment is a potential high-risk window.

## 4 Discussions

This study conducted a pharmacovigilance evaluation utilizing a large sample of FAERS data to systematically compare the ocular safety profiles of three major long-acting basal insulins. Data mining results revealed characteristic differences in association strength, event phenotypes, and time-to-onset patterns among the three medications, providing references for clinical drug safety monitoring.

### 4.1 Differences in association strength and potential mechanisms

Data revealed differences in the association strength between the three medications and visual impairment: insulin glargine > insulin detemir > insulin degludec. This divergence may primarily stem from their differences in molecular engineering and pharmacokinetic profiles.

Insulin glargine has an isoelectric point of pH 4.0. Upon subcutaneous injection into a neutral physiological environment (pH 7.4), it undergoes microprecipitation to form a depot (13, 14). This alteration in the local microenvironment, combined with its 24-hour action profile, may have certain impacts on vascular tension within the retina. Relevant pharmacological studies indicate that insulin glargine has a higher binding affinity for the IGF-1 receptor—up to 6 to 8 times that of native human insulin (2). The activation of IGF-1 receptors has mitogenic effects, which may to some extent affect ocular microvascular proliferation, thereby increasing the potential risk of visual impairment.

Conversely, insulin degludec is modified via fatty acid acylation to form stable, soluble multi-hexamers in the subcutaneous tissue. Its molecular structure more closely resembles physiological insulin, and its pharmacokinetic profile is relatively stable without distinct peaks, theoretically causing less interference to the ocular microenvironment (15). Insulin detemir reversibly binds to albumin via an aliphatic fatty acid chain, providing an intermediately stable profile (16), which aligns with the intermediate risk signal observed in this study. Therefore, in clinical practice, when treating diabetic patients with severe pre-existing ocular conditions, clinicians may consider these pharmacological characteristics comprehensively during drug selection.

### 4.2 Event specificity: retinopathy vs. glaucoma

This study observed that different insulins might be associated with specific ocular phenotypes. Insulin degludec and insulin detemir predominantly demonstrated safety signals associated with diabetic retinopathy. Mechanistically, this might be related to minor fluctuations in their plasma concentrations. Such slight glycemic variations, particularly when postprandial control might be slightly inferior to that of insulin glargine, could stimulate the expression of local Vascular Endothelial Growth Factor (VEGF), subsequently affecting retinal endothelial function (16–18).

In comparison, insulin glargine exhibited a relatively prominent signal association with diabetic glaucoma (ROR = 50.36). This may be related to its stronger affinity for the IGF-1 receptor. Previous studies indicate that IGF-1 receptors are highly expressed in human trabecular meshwork cells (19, 20). The potential long-term effects of insulin glargine on these receptors could influence extracellular matrix remodeling within the trabecular meshwork, progressively increasing aqueous humor outflow resistance. These alterations in intraocular pressure regulation may play a certain role in the onset or progression of secondary neovascular glaucoma, a late-stage complication of diabetic eye disease (21).

### 4.3 Interpretation of the “body weight” factor: clinical confounding, pathological differences, and reporting bias

The multivariate regression model suggested that higher body weight might statistically act as a “protective factor” against visual impairment. From a pharmacokinetic perspective, this has been hypothetically attributed to a “subcutaneous fat buffering effect” (22–24), where a thicker subcutaneous fat layer might where a thicker subcutaneous fat layer might slow insulin absorption and dampen abrupt fluctuations in plasma insulin levels, thereby mitigating the potential damage of rapid glycemic swings to retinal microvasculature.

However, from an epidemiological and clinical perspective, this statistical result must be interpreted with extreme caution, as it is highly susceptible to confounding by indication. The FAERS database cannot effectively distinguish between patients with Type 1 Diabetes Mellitus (T1DM) and Type 2 Diabetes Mellitus (T2DM). In clinical settings, T1DM patients are typically leaner (lower body weight), have an earlier age of onset, are absolutely dependent on insulin, and due to a longer disease duration, generally possess a higher baseline risk for microvascular complications, including retinopathy. Conversely, a larger proportion of T2DM patients are overweight or obese (higher body weight), and the initial progression of their ocular complications is often relatively slower.

Furthermore, it is crucial to emphasize that our data extraction revealed an exceptionally high proportion of missing baseline body weight data across all three insulin cohorts (missing rates: 88.4% for Degludec, 76.8% for Detemir, and 91.5% for Glargine). This massive data sparsity introduces substantial reporting bias, further limiting the reliability of this specific variable.

Therefore, the higher body weight “protective” signal observed in this study’s data is severely limited by data missingness and is more likely a statistical proxy reflecting the less aggressive pathophysiological trajectory of T2DM compared to T1DM, rather than indicating that obesity itself provides substantive biological protection. The general consensus in the medical community remains that obesity exacerbates insulin resistance and increases the risk of various systemic complications, including nephropathy and cardiovascular diseases (25–27).

### 4.4 Temporal patterns and early worsening of diabetic retinopathy

The Weibull distribution analysis indicated that all three insulins conformed to the “early failure model,” with insulin degludec showing a shorter median onset time (23.6 days). This temporal distribution characteristic is consistent with the clinical phenomenon known as “Early Worsening of Diabetic Retinopathy” (EWDR) (28, 29).

When diabetic patients remain in a chronic hyperglycemic state, the osmotic pressure within the ocular lens and the aqueous humor reaches a pathological equilibrium. Upon the initiation of basal insulin therapy, blood glucose levels often decline rapidly. This systemic glycemic correction may temporarily disrupt the intraocular osmotic balance, causing water to enter the lens. Transient lens edema induced by this osmotic alteration can present clinically as early-stage visual blurriness or visual impairment. As the body gradually adapts to the normalized glycemic environment, the osmotic pressure typically re-equilibrates, and acute visual symptoms may spontaneously resolve. This mechanism explains why the incidence rate of visual impairment is relatively higher in the initial weeks of treatment and progressively decreases over long-term therapy.

### 4.5 Limitations

As a pharmacovigilance study based on a spontaneous reporting system, this study has the following limitations:

First, FAERS relies on voluntary reporting, which inevitably introduces reporting bias and underreporting phenomena.

Second, the database does not provide denominator data such as prescription volume or Defined Daily Doses (DDD). Consequently, the large report volume of insulin glargine (n=83,680) is partially influenced by the “Weber effect,” its longer time on the market, and its high market share; thus, report volumes and ROR values cannot be directly equated with true clinical incidence rates.

Third, FAERS lacks certain critical baseline clinical data, including HbA1c levels, fasting blood glucose, exact diabetes duration, and detailed prior ophthalmic medical history. This makes it difficult to completely distinguish drug-induced adverse events from the natural progression of diabetic eye disease. Furthermore, the high missing rate of demographic data (especially body weight) limited the conduct of further sensitivity analyses.

Finally, the vast majority of data in this study originated from the United States (>93% of reports). To verify the generalizability of the results, queries were attempted in the Japanese Adverse Drug Event Report (JADER) database and the Canada Vigilance Adverse Reaction Database (CVARD); however, the number of specific ocular adverse event reports related to these three insulins in these regional databases was too small to conduct a comparative analysis with sufficient statistical power. Therefore, the findings of this study primarily establish statistical associations of disproportionate reporting rather than definitive causal relationships.

## 5 Conclusion

In summary, long-acting basal insulins are an important modality in diabetes treatment, but their use exhibits statistically significant safety signal associations with visual impairment. Through the mining of the FAERS database, this study observed that insulin glargine presented a relatively stronger overall risk signal, primarily manifesting as an association with diabetic glaucoma; meanwhile, insulin degludec and insulin detemir showed relatively weaker signals, mainly associated with diabetic retinopathy. Additionally, these visual impairment events demonstrated an “early-onset” temporal pattern, concentrating primarily within the first few weeks to months after therapy initiation.

These results suggest that in clinical practice, healthcare institutions may consider implementing individualized ophthalmic monitoring strategies during the early phase of initiating basal insulin therapy—for example, conducting intraocular pressure screening for patients using insulin glargine, and fundus examinations for those using insulin degludec and insulin detemir. Given the inherent limitations of spontaneous reporting systems, well-designed, large-scale prospective cohort studies are needed in the future to further validate these safety signals and elucidate their underlying mechanisms.

## Data Availability

The original contributions presented in the study are included in the article/supplementary material. Further inquiries can be directed to the corresponding author.
